# A comparative study of two-dimensional and three-dimensional ultrasonography in evaluation of gastric affections in dogs

**DOI:** 10.14202/vetworld.2015.707-712

**Published:** 2015-06-06

**Authors:** Madan Pal, Prem Singh, Rishi Tayal, Dinesh Dehmiwal, S. M. Behl, Sarvan Kumar, R. K Chandolia

**Affiliations:** 1 Department of Veterinary Surgery and Radiology, Lala Lajpat Rai University of Veterinary & Animal Sciences, Hisar, Haryana, India; 2 Department of Veterinary Pathology, Lala Lajpat Rai University of Veterinary & Animal Sciences, Hisar, Haryana, India; 3 Department of Veterinary Gynaecology and Obstetrics, Lala Lajpat Rai University of Veterinary & Animal Sciences, Hisar, Haryana, India

**Keywords:** dogs, gastritis, gastric dilatation, gastric foreign bodies, gastric ulcer, three-dimensional ultrasonogram

## Abstract

**Aim:**

The objective of the study was to obtain and compare the two-dimensional (2D) and three-dimensional (3D) ultrasonographic images of pathological conditions of the stomach in dogs in clinical cases.

**Materials and Methods:**

In our study, 12 clinical conditions of the stomach were recorded using ultrasonography. The ultrasound machine used for this study was 3D ultrasound machine (Nemio-XG: Toshiba, Japan) having four-dimensional volumetric transducer.

**Results:**

Present study was done to compare 2D and 3D ultrasonographic images in different gastric affections in dogs. In case of uremic gastropathy due to inflammatory response, the wall of the stomach was 0.6 cm thick and hyperechoic and gastric folds were also hyperechoic indicative of gastritis. In second, third, and fourth case of gastritis the wall of the stomach was 0.7, 0.6, and 0.55 cm, respectively thick and hyperechoic. In fifth and sixth case of gastritis, inflammatory response due to ingestion of polythene and sand led to gastritis and ultrasonographically, the wall of the stomach was 0.6 cm and 0.7 cm thick, respectively, and hyperechoic. In case of gastric ulcer, ultrasonographically, there was a disruption of gastric mucosal layer. In cases of gastric dilatation, anechoic content indicating fluid was seen in stomach area and due to dilatation boundary of the stomach was not clear and the increase in the lumen of the stomach was observed. In case of foreign body, ultrasonographically the wall of the stomach was 0.55 cm thick and hyperechoic. In the middle of the stomach, multiple hyperechoic shadows of the foreign bodies i.e. leather and bunch of straw of grass were observed. In case of pyloric stenosis ultrasonographically, anechoic lumen of the pylorus surrounded by 0.5 cm hypoechoic thickened muscle. In some cases, 3D ultrasonography was not diagnostic i.e. gastric foreign bodies and gastric dilatation. These conditions were better visualized on the 2D sonogram.

**Conclusion:**

The appearance of clinical conditions of the stomach such as gastritis and pyloric stenosis were more distinct on 3D ultrasonogram than 2D ultrasonogram. The 3D ultrasonography was not diagnostic in cases of gastric foreign bodies and gastric dilatation.

## Introduction

Abdominal ultrasonography provides valuable information that led to a definitive diagnosis or to narrow the list of differential diagnosis obtained with other diagnostic techniques [1]. The ultrasound of stomach was initially performed to detect the organic disease of the gastric wall but later on different methods have been developed to study a functional aspect of gastric pathology. Ultrasound can be used to evaluate antral contractibility, gastric emptying, transpyloric flow, gastric configuration, intragastric distribution of the meal, gastric accommodation, and strain measurement of the gastric wall. However, nowadays advanced method for three-dimensional (3D) ultrasound imaging have also been developed to study diseases of stomach [2].

Ultrasonography is more sensitive than radiographic survey for the identification of gastric lesions in dogs. Gastrointestinal (GI) foreign bodies greatly vary in size, shape, and echogenicity. The fluid or gas accumulation within the stomach or part of the intestinal tract is an indicator of mechanical ileus or obstruction. Balls are easily identified because of their characteristic curvilinear interface. Linear foreign bodies present as bright linear interfaces [1].

The actual cause of the obstruction may be better visualized on ultrasound examination than abdominal radiographs. Pyloric outflow obstruction, especially if chronic, usually results in fluid distension of the gastric lumen. The fluid enhances visualization of the pylorus and any potential foreign bodies or wall thickening [1]. The wall thickening is the most common finding in inflammatory diseases. The GI neoplasia is often associated with motility disturbances that produce luminal fluid accumulation, which optimizes visualization of the lesion. Complete loss of visualization of wall layering is common with gastric lesion and is considered the most specific ultrasound indication of neoplastic condition [3]. The ultrasonographic images of gastritis resulting in accumulation of gases, fluid, and various kinds of foreign bodies are different. Therefore, it is required that two-dimensional (2D) ultrasonographic images of fluid, foods, and different type of foreign bodies should be studied and compared with 3D ultrasonographic images. The radiographic images of the foreign body or any other abnormality are taken in the routine manner but ultrasonographic images depending upon the gastric pathology especially 3D images of the stomach abnormalities are not available. Ultrasonographic examination of the GI tract is often challenged by the presence of gas and/or feces in the stomach and intestine.

2D ultrasonography of the stomach stands out as a simple and well-validated means by which antral area, proximal stomach accommodation and gastric emptying can be assessed, whereas 3D ultrasonography offers added information about the physiology of the stomach [4]. The use of ultrasound to assess the antrum is appealing in both the clinical and research setting due to the ease and simplicity with which it can be applied, especially in favor of other imaging modalities that are more expensive and time consuming [4]. In veterinary clinical medicine, ultrasonography helps in detecting pathological abnormalities of the stomach and also to evaluate the gastric lesions [5].

Aim of the present study was to compare two-dimensional and three-dimensional ultrasonographic images of different gastric affections in dogs.

## Materials and Methods

### Ethical approval

The study was conducted after the approval of the Institutional Animal Ethics Committee.

### Study area

The study was conducted in the Department of Veterinary Surgery and Radiology with the collaboration of Department of Veterinary Gynaecology and Obstetrics, College of Veterinary Sciences, Lala Lajpat Rai University and Animal Sciences, Hisar (Haryana).

### Animals

The 12 dogs reported to Teaching Veterinary Clinical Complex College of Veterinary Sciences, Lala Lajpat Rai University and Animal Sciences, Hisar (Haryana) used for the study of pathological conditions of the stomach.

### Ultrasonographic examination

Scanning area was shaved properly and enough ultrasound gel was applied over the site and the surface of the transducer to have better skin transducer contact to get a better image. The dogs were sedated with xylazine @ 1 mg/kg body weight for restraining. The ultrasound machine used for this study was 3D ultrasound machine (Nemio-XG: Toshiba, Japan) having four-dimensional (4D) volumetric transducer. The images were acquired with 3-6 MHz 2D curvilinear transducer and 4.2-6 MHz 4D volumetric curvilinear transducer operated at 3-5 MHz frequency. For scanning of stomach, the animals were kept in right lateral recumbency keeping in view the topographic position of the stomach just below the left rib cage. For scanning, the curvilinear probe was placed below the left rib cage. After completing the cross-sectional imaging of the stomach, the transducer is turned 90° to evaluate in the longitudinal plane [6]. The canine stomach can be accessed immediately caudal to the liver, at the level of the xiphoid [3]. The canine stomach is centrally placed and the long axis is essentially perpendicular to the vertebral column, so the longitudinal imaging planes allow cross-sectional images of the stomach [6]. Fasting for a period of time prior to the examination will minimize the fluid and air present in the stomach resulting in better quality images [7].

## Results

In our study, 12 clinical conditions of the stomach were recorded using ultrasonography. Out of which six cases of gastritis, one case of gastric ulcer, three cases of gastric dilatation, one case of gastric foreign body, and one case of pyloric stenosis.

**Figure-1:**
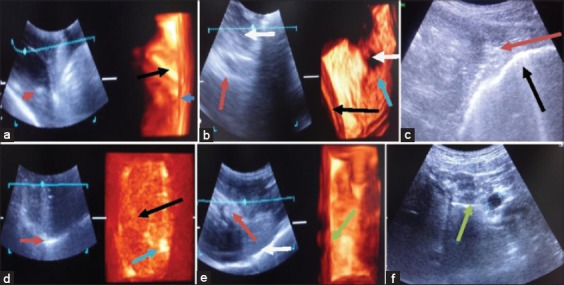
(a) Sonogram of stomach in first case of gastritis thickening of gastric wall (blue arrow). Gastric folds are hyperechoic (red arrow in two-dimensional [2D] sonogram and black arrow in three-dimensional [3D] sonogram), (b) sonogram of stomach in second case of gastritis thickening of gastric wall (red arrow in 2D sonogram and black arrow in 3D sonogram), (c) sonogram of stomach in third case of gastritis thickening of gastric wall (black arrow), (d) sonogram of stomach fourth case of gastritis thickening of gastric wall (red arrow in 2D sonogram), (e) sonogram of stomach in fifth case of gastritis thickening of gastric wall (white arrow in 2D sonogram). Gastric fold are hyperechoic (red arrow in 2D sonogram and green arrow in 3D sonogram), (f) sonogram of stomach in sixth case of gastritis thickening of gastric wall (green arrow).

First case of gastritis dog was a male of 6 years old was reported in TVCC, Hisar with the history of urinary incontinence since 10 days. The dog was vomiting simultaneously also. In 2D ultrasonogram, the gastric wall was 0.6 cm thick and hyperechoic. There were different shadows of stomach lumen depending on the presence of gastric contents. Toward caudal and cranial side, there was anechoic content representing fluid in the stomach. Toward ventral side, the image was hypoechoic with echogenic striations due to the presence of semisolid food. The gastric fold was appreciated as hyperechoic striations. The thickening of gastric wall and prominence of gastric folds are indicative of gastritis (Figure-1a). In 3D ultrasonogarm, the gastric fold was more distinct and clear. The prominence of the gastric fold is due to inflammatory response. The anechoic content of the fluid toward caudal side is also more distinct on 3D ultrasonogram (Figure-1a). The gastric wall was more prominent on 3D ultrasonogram. Different layers of the gastric wall which were not clear on 2D ultrasonogram were seen separately on 3D ultrasonogram. The four layers of the stomach i.e. mucosa, submucosa, muscularis, and serosa were identified on 3D ultrasonogram. The thickening of gastric wall and appearance of separate layers along with the prominence of gastric fold was indicative of gastritis. The inflammatory changes were more distinct on 3D ultrasonogram (Figure-1a). Second case of gastritis of a female dog of 3 years was reported in TVCC, Hisar with a history of abdominal distension, vomition, and anorexia since a month. In 2D ultrasonogram, the gastric wall was 0.7 cm thick and hyperechoic. In 2D ultrasonogram, the cavity of the stomach was full of food. Toward dorsal side, there was an anechoic shadow of the fluid. Toward ventral side, there was uniform hypoechoic shadow of the semisolid food. Thickening of gastric wall was indicative of the gastritis (Figure-1b). These images were more distinct on 3D ultrasonogram. In 3D ultrasonogram, more distinct thickened gastric wall was seen with all four separate layers of the stomach, i.e., mucosa, submucosa, muscularis, and serosa. The anechoic and hypoechoic shadow of the stomach contents were clearer on 3D ultrasonogram. The inflammatory changes were more distinct on 3D ultrasonogram (Figure-1b). Third case of gastritis of a male dog of 2 years was reported in TVCC, Hisar with a history of fever, vomition, inappetence, and weight loss since 1 month. In 2D ultrasonogram, gastric wall was 0.6 cm thick and hyperechoic. The hypoechoic shadow of the semisolid food particle and hyperechoic shadow of solid food particle was seen in 2D ultrasonogram. The gastric folds were not seen in 2D ultrasonogram. Toward ventral side of stomach hyperechoic reflection of the gas was present in 2D ultrasonogram. In 2D ultrasonogram, thickening of the gastric wall was indicative of the gastritis (Figure-1c). Fourth case of a male dog of 5 years with a history of ingestion of polythene bag and not passing feces since 7 days. In 2D ultrasonogram toward cranial side of the stomach, there was appearance of anechoic fluid in lumen of the stomach. The gastric wall appeared hyperechoic and 0.55 cm thick. The hypoechoic shadow of the semisolid food particles and hyperechoic shadow of solid food particle was seen on 2D ultrasonogram. The ultrasonographic image of polythene could not be visualized. Due to ingestion of polythene there was tendency of vomiting (Figure-1d). In 3D ultrasonogram, the hypoechoic shadow of the semisolid food particles and hyperechoic shadow of solid food particle was clearly seen (Figure-1d). Fifth case of gastritis of a male dog of 6 months year was reported in TVCC, Hisar with history of vomition and anorexia since 10 days. In 2D ultrasonogram, gastric wall was 0.6 cm thick and hyperechoic. The anechoic shadow of fluid in the lumen of stomach was seen in 2D ultrasonogram. The semisolid gastric contents appear as hypoechoic in 2D ultrasonogram. The gastric fold was appreciated as hyperechoic striations. The thickening of gastric wall and prominence of gastric folds are indicative of gastritis (Figure-1e). The gastric wall was more prominent on 3D ultrasonogram. Different layers of gastric wall, which were not clear on 2D ultrasonogram were seen separately on 3D ultrasonogram. The anechoic shadow of fluid toward dorsal and caudal side of the stomach was prominent in 3D ultrasonogram. The thickening of gastric wall and appearance of separate layers along with prominence of gastric fold was indicative of gastritis. The inflammatory changes were more distinct on 3D ultrasonogram (Figure-1e). Sixth case of gastritis of a male dog of 18 months old was reported with a history of eating inanimate objects since 1 month. Ultrasonographically, the hyperechoic shadow of solid food particle and hypoechoic shadow of semisolid food particle was seen in lumen of the stomach. The gastric wall was 0.7 cm thick and hyperechoic indicative of gastritis in the dog. The anechoic shadow of fluid toward ventral side of the stomach was also visible (Figure-1f).

### Gastric ulcer

**Figure-2:**
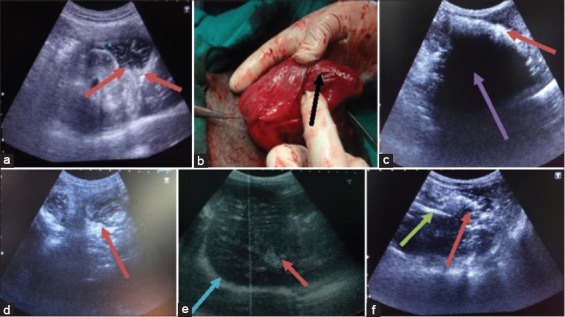
(a) Two-dimensional (2D) sonogram in case of gastric ulcer, thickening of gastric wall (black arrow) and disruption of mucosal layer is hyperechoic (red arrow), (b) gastric lesion on mucosa of stomach as gastric ulcer, (c) 2D sonogram in first case of gastric dilatation, there is anechoic fluid (blue arrow) in lumen of stomach due to whick boundaries are not clear, (d) 2D sonogram in second case of gastric dilatation the solid food in lumen of stomach is hyperechoic (red arrow) and there is dilatation of stomach due to which boundaries of stomach is not clear, (e) 2D sonogram in third case of gastric dilatation, there is shadow of full sized stomach due to dilatation with the solid food in lumen of stomach is hyperechoic (red arrow) and thickening of gastric wall as hyperechoic shadow (blue arrow), (f) 2D sonogram of gastric foreign bodies, there is hyperechoic shadow of bunch of straw (red arrow) and leather piece (green arrow) in lumen of stomach.

A dog of 5 years old was brought to TVCC with a history of eating some pieces of bone and blood in vomition since 1 month. No radiographic lesions were noted on the radiograph. In 2D ultrasonography, stomach wall was hyperechoic. In lumen of stomach, anechoic shadow of fluid and hyperechoic shadow of solid food particle was seen. In 2D ultrasonogram, there was disruption of mucosal layer of the stomach and a localized hyperechoic shadow was seen that was indicative of gastric ulcer (Figure-2a). Gastrotomy was done under general anesthesia and gastric lesions were found on mucosa of stomach diagnosed as gastric ulcer (Figure-2b).

### Gastric dilatation

First case of a male dog was reported with a history of abdominal distension, salivation, and vomiting (retching with inability to vomit) since 15 days. In 2D ultrasonogram, the boundaries of the stomach were not imaged due to dilatation. In the middle of the stomach, anechoic shadow of fluid was seen. On dorsal side of stomach, hyperechoic shadow of the solid food was present. Stomach wall was not be visualized (Figure-2c). In this case, 3D ultrasonogram was not diagnostic. Second case of gastric dilatation of a male dog of 1-year was reported with a history of anorexia, vomition, and pot-bellied. In laboratory findings, dog was anemic with hemoglobin 8.2 g% and a slight increase in neutrophiles. Ultrasonographically, the gastric wall was not be visualized on 2D ultrasonogram. Different types of ultrasonographic images of food contents were seen. In the middle of the stomach, hyperechoic image of mass of solid food was visible while toward caudal side anechoic image of the fluid was present. Toward ventral side, hypoechoic image of the food was seen (Figure-2d). Third case of gastric dilatation of a male dog of 7 year was reported with a history of abdominal distension, anorexia, vomition since 3 months. In 2D ultrasonogram, thick gastric wall appears hyperechoic. The anechoic shadow of fluid in the lumen of the stomach was seen in 2D ultrasonogram. The solid gastric content appears hyperechoic and semisolid gastric contents appear as hypoechoic throughout the lumen of the stomach. Increase in lumen of the stomach was indicative of the dilatation of the stomach which was externally manifested by pot-bellied appearance (Figure-2e).

### Gastric foreign body

Case of a female dog of 8 month was reported with a history of vomition since 1 month and sign of abdominal pain. In 2D ultrasonogram wall of the stomach was 0.55 cm thick and hyperechoic. There were multiple images of food particles could be seen in the lumen of the stomach. Toward ventral side of stomach anechoic shadow of fluid in lumen of the stomach was seen. In the middle of the stomach, there were multiple hyperechoic shadows of foreign bodies were visible (Figure-2f). Theses shadows were of leather piece and bunch of straws of the grass as revealed on surgical intervention. On basis of ultrasonographic diagnosis, gastrotomy was done under general anesthesia and foreign bodies like leather piece, bunch of grass straw, and plastic piece were removed from the stomach of dog.

### Pyloric stenosis

**Figure-3:**
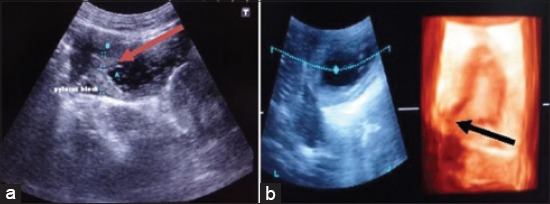
(a) Two-dimensional sonogram of pyloric stenosis, there is hyperechoic thickening of muscle of pyloric antrum (red arrow), (b) three-dimensional sonogram of pylori stenosis, there is hyperechoic thickening of muscle of pylori antrum (black arrow).

A case of 5 years old dog was brought to TVCC with a history of eating some pieces of bone and blood in vomition and abdominal distension since a month. Ultrasonographically, thickening of pyloric antrum was seen in 2D ultrasonogram. The image of the 0.5 cm thickened muscles of the pylorus antrum was hypoechoic (Figure-3a). The anechoic lumen of the pylorus was seen as fine streak surrounded by 0.5 cm thick muscles. Due to narrowing of the pylorus there was accumulation of fluid in the stomach. There was accumulation of fluid marked by anechoic shadow with mixing of intermittent solid echogenic particles. On 3D ultrasonogram, the narrowing of pylorus was distinct as compared to 2D ultrasonogram (Figure-3b).

## Discussion

Ultrasonography is a useful aid to abdomen for evaluation of the GI tract. It has been used to detect and evaluate the foreign bodies such as rubber ball, leather, grass, etc., [8] and tumor. Gas can create artifacts and may completely obscure the wall of the stomach.

In our study 12 clinical conditions of the stomach were recorded using ultrasonography. Out of which six cases of gastritis, one case of gastric ulcer, three cases of gastric dilatation, one case of gastric foreign body, and one case of pyloric stenosis. Ultrasonography is more sensitive than radiography for the identification of gastric lesions in different conditions like gastric ulcer and gastritis in dogs [2]. In our study, out of six cases of gastritis one case was of uremic gastropathy. In this case in 2D ultrasonogram, the gastric wall was 0.6 cm thick and hyperechoic. The gastric folds were appreciated as hyperechoic striations. The thickening of gastric wall and prominence of gastric folds were indicative of gastritis. In 3D ultrasonogarm, the gastric folds were more distinct and clear. The prominence of gastric fold was due to inflammatory response. Grooters *et al*. [9] also observed that gastritis occurs commonly in the patients with chronic uremia. The ultrasonographic features were moderately thickened gastric wall with prominent rugal folds and a hyperechoic line at the mucosal luminal interface secondary to mineralization of the mucosa. Acute gastritis is the most common cause of acute vomiting in the dogs. In our study, three cases of vomiting dogs were reported. In all three cases in 2D ultrasonogram, gastric wall was 0.7 cm, 0.6 cm and 0.6 cm thick respectively and hyperechoic. Thickening of the gastric wall was indicative of the gastritis. The gastritis may also occur in cases of ingestion of inanimate objects. In our study, two cases were observed. One case was reported with the history of ingestion of polythene bag since 7 days. Due to ingestion of polythene there was tendency of vomiting and cause gastritis. The gastric wall was 0.55 cm thick and hyperechoic. The thickening of gastric wall was indicative of gastritis. The second case was reported with the history of ingestion of sand since 1 month. The gastric wall was 0.7 cm thick and hyperechoic. The anechoic shadow of fluid in the stomach was also visible. The thickening of gastric wall was indicative of gastritis. The layers were maintained with thickening of the mucosa. Mittelstaedt [10], reported that acute or severe inflammation can cause a uniform thickening of the gastric wall and chronic gastritis may result in localized or diffuse thickening of gastric wall. Penninck *et al*. [11], also measured the normal stomach wall thickness in dogs and were in range of 0.2-0.5 cm.

In one case, there was a history of hematemesis. In this case of gastric ulcer 2D ultrasonogram, showed thickening of gastric wall and the gastric wall was hyperechoic. There was disruption of mucosal layer of stomach and a localized hyperechoic shadow, which was indicative of gastric ulcer. Penninck *et al*. [12], also observed gastric ulcer with the help of ultrasonographically, there was loss of all the five layers of stomach and accumulation of the fluid in the lumen of the stomach.

Three cases of gastric dilatation were observed. Gastric dilatation means dilatation of the stomach may be due to the accumulation of gas and/or fluid. Any disturbance in feeding schedule can cause dilatation of the stomach. Heavy diet and lack of exercise can also cause dilatation of the stomach. First case was reported with a history of abdominal distension, salivation, and vomiting (retching with inability to vomit) since 15 days. Ultrasonographically, in the middle of the stomach anechoic shadow of fluid was seen. The boundaries of the stomach could not be imaged due to dilatation. Second case was reported with of history anorexia, vomition, and pot-bellied. The gastric wall could not be visualized on 2D ultrasonogram due to dilatation. Third case was reported with a history of abdominal distension, anorexia, vomition since 3 months. Ultrasonographically, there was an anechoic shadow of fluid in the lumen of stomach. Increase in lumen of the stomach was indicative of the dilatation of the stomach which was externally manifested by pot-bellied appearance.

In our study, one case of gastric foreign body was observed. A female dog of 8 months was reported with a history of vomition since 1 month and sign of abdominal pain. In 2D ultrasonogram, wall of the stomach was 0.55 cm thick, and hyperechoic. In the middle of the stomach, there were multiple hyperechoic shadows of foreign bodies were visible.Theses shadows were of leather piece and bunch of straws of the grass as revealed on surgical intervention. Tidwell and Penninck [8], observed that the presence of bright interface associated with strong shadowing was indicative of foreign material.

In our study, one case of pyloric stenosis was observed. The image of 0.5 cm thickened muscles of the pylorus antrum was hypoechoic. The anechoic lumen of the pylorus could be seen as fine streak surrounded by thick muscles. There was an accumulation of fluid marked by anechoic shadow with mixing of intermittent solid echogenic particles in the stomach. Spevak *et al*. [13], also observed that thickened muscular layer appeared as hypoechoic on transverse imaging of pylorus. Haller and Cohen [14], ultrasonographically observed that the muscular layer was hypoechoic and 4 mm thick or large.

## Conclusion

In conclusion, result of the current study showed that the appearance of clinical conditions of the stomach such as gastritis and pyloric stenosis were more distinct on 3D ultrasonogram than 2D ultrasonogram. The 3D ultrasonography was not diagnostic in cases of gastric foreign bodies and gastric dilatation.

## Authors’ Contributions

MP and PS have designed the study and planned the research experiment. MP performed the research experiments. PS, RT, SMB, and RKC supervised the research. DD and SK helped in conducting experiments. All the authors read and approved the final manuscript.

## References

[1] Gomaa M, Samy M.T, Omar M.S.A, Mekkawy N.H (2012). Ultrasonographic findings of most common surgical disorders of gastrointestinal tract in dogs and cats. Iran. J. Vet. Surg.

[2] Gilja O.H (2007). Ultrasound of the stomach. Ultraschall, Med.

[3] Larson M.M, Biller D.S (2009). Ultrasound of gastrointestinal tract. Vet. Clin. Small Anim.

[4] Trahair L, Jones K.L (2012). Ultrasonography of the stomach. Applied Aspects of Ultrasonography in Humans. InTech, Rijeka, Croatia.

[5] Choi M, Seo M, Jung J, Lee K, Yoon J, Chang D, Park R.D (2002). Evaluation of canine gastric motility with Ultrasonography. J. Vet. Med. Sci.

[6] Larson M.M (2013). Ultrasound of the Vomiting Dog. Western Veterinary Conference.

[7] Nylund K, Odegaard S, Hausken T, Folvik G, Lied G.A, Viola I, Hauser H, Gilja O.H (2009). Sonography of the small intestine. World J. Gastroenterol.

[8] Tidwell A.S, Penninck D.G (1992). Ultrasonography of gastrointestinal foreign bodies. Vet. Radiol. Ultrasound.

[9] Grooters A.M, Miyabayashi T, Biller D.S, Merryman J (1994). Sonographic appearance of uremicgastropathy in four dogs. Vet. Radiol. Ultrasound.

[10] Mittelstaedt C.A (1992). Gastrointestinal tract. General Ultrasound.

[11] Penninck D.G, Nyland T.G, Fisher P.E, Kerr L.Y (1989). Ultrasonography of the normal canine gastrointestinal tract. Vet. Radiol.

[12] Penninck D, Matz M, Tidwell A (1997). Ultrasonography of gastric ulceration in the dog. Vet. Radiol. Ultrasound.

[13] Spevak M.R, Ahmadjian J.M, Kleinman P.K (1992). Sonography of hypertrophic pyloric stenosis:Frequency and cause of non-uniformechogenecity of the thick-ended pyloric muscle. Am. J. Roentgenol.

[14] Haller J.O, Cohen H.L (1986). Hypertrophic pyloric stenosis:Diagnosis using US. Radiol.

